# Disentangling environmental correlates of vascular plant biodiversity in a Mediterranean hotspot

**DOI:** 10.1002/ece3.762

**Published:** 2013-09-15

**Authors:** Rafael Molina-Venegas, Abelardo Aparicio, Francisco José Pina, Benito Valdés, Juan Arroyo

**Affiliations:** Departamento de Biología Vegetal y Ecología, Universidad de SevillaApartado 1095, E-41080, Sevilla, Spain

**Keywords:** Alpha and beta diversity, Baetic-Rifan hotspot, elevation, endemism, lithology, Mediterranean flora

## Abstract

We determined the environmental correlates of vascular plant biodiversity in the Baetic-Rifan region, a plant biodiversity hotspot in the western Mediterranean. A catalog of the whole flora of Andalusia and northern Morocco, the region that includes most of the Baetic-Rifan complex, was compiled using recent comprehensive floristic catalogs. Hierarchical cluster analysis (HCA) and detrended correspondence analysis (DCA) of the different ecoregions of Andalusia and northern Morocco were conducted to determine their floristic affinities. Diversity patterns were studied further by focusing on regional endemic taxa. Endemic and nonendemic alpha diversities were regressed to several environmental variables. Finally, semi-partial regressions on distance matrices were conducted to extract the respective contributions of climatic, altitudinal, lithological, and geographical distance matrices to beta diversity in endemic and nonendemic taxa. We found that West Rifan plant assemblages had more similarities with Andalusian ecoregions than with other nearby northern Morocco ecoregions. The endemic alpha diversity was explained relatively well by the environmental variables related to summer drought and extreme temperature values. Of all the variables, geographical distance contributed by far the most to spatial turnover in species diversity in the Baetic-Rifan hotspot. In the Baetic range, elevation was the most significant driver of nonendemic species beta diversity, while lithology and elevation were the main drivers of endemic beta diversity. Despite the fact that Andalusia and northern Morocco are presently separated by the Atlantic Ocean and the Mediterranean Sea, the Baetic and Rifan mountain ranges have many floristic similarities – especially in their western ranges – due to past migration of species across the Strait of Gibraltar. Climatic variables could be shaping the spatial distribution of endemic species richness throughout the Baetic-Rifan hotspot. Determinants of spatial turnover in biodiversity in the Baetic-Rifan hotspot vary in importance between endemic and nonendemic species.

## Introduction

In recent decades, the detailed description and understanding of the distribution of biodiversity on Earth has become one of the most important challenges for ecologists, taxonomists, and evolutionary and conservation biologists. According to Myers et al. (2000), an extraordinarily small proportion of the Earth's land surface harbors a large part of the world's plant biodiversity: about 44% of all vascular plant species worldwide are to be found on just 1.4% of the Earth's surface area. These extraordinarily species-rich areas are of special interest when prioritizing conservation actions since they act as reservoirs for plant biodiversity (Myers 1999). The Mediterranean Basin is one such biodiversity hotspots, mainly due to its high speciation (Cowling et al. 1996) and low extinction rates (Rodríguez-Sánchez et al. 2008). It accounts for 4.3% of all extant vascular plant species (but see Médail and Quézel 1997 for a higher estimate) and is only surpassed in this sense by the tropical Andes and the Sundaland biogeographical region in South-East Asia (Myers et al. 2000).

Despite some important advances, much still remains to be carried out to bridge the gap between extensive accounts at world or regional scales and detailed insights into the environmental drivers of biodiversity patterns in particular species groups and regions. Although the number of plant groups used as study cases for a given biodiversity hotspot is rapidly increasing (e.g., Ojeda 1998; Mansion et al. 2008; Santos-Gally et al. 2012), regional accounts and their environmental correlates are less commonly conducted for entire hotspots (an important exception is that of Cowling et al. 1992 for the Cape Floristic Region). Factors such as a lack of taxonomic knowledge of the biota and poor biogeographical data from naturally defined areas (as opposed to political or cultural territories) are in part responsible for this situation. Moreover, most of the descriptive accounts of biodiversity are based on estimations of alpha diversity at different scales, from whole regions down to areas nested within these much larger regions (Cowling et al. 1992). Despite the fact that the environments and species diversity in most biodiversity hotspots are not homogeneous, less attention has been paid to patterns of species turnover in areas with contrasting environments (Cowling and Lombard 2002). Heterogeneity within hotspots, on the other hand, offers an excellent opportunity for understanding the environmental factors that generate and maintain patterns of biodiversity.

Evidence that the great floristic richness of the Mediterranean Basin is unevenly distributed is provided by the high species turnovers found along sharp environmental gradients (Thompson 2005) and the great richness in narrow endemic species (Médail and Quézel 1999). Thus, it is crucial that we document how environmental gradients shape Mediterranean species assemblages to better understand the origin of Mediterranean floristic diversity and improve our power to predict its fate in light of global changes. Nevertheless, unlike the situation in other Mediterranean-type climate regions, the analysis of species turnover in the Mediterranean Basin has to date received very little attention (Cowling et al. 1994; Cowling and Lombard 2002; Figueroa et al. 2011; Sander and Wardell-Johnson 2011).

Although close to 60% of all native species in the Mediterranean are endemic to this region as a whole (Greuter 1991), a large majority of these endemic species are confined to a given region. By contrast, only 28% of nonendemic species occur in a single region in the Mediterranean (Thompson 2005). This remarkable difference in the overall distribution pattern of narrow endemic and nonendemic Mediterranean species would seem to indicate that determinants of spatial biodiversity distribution in the Mediterranean Basin differ (or vary in their strength or importance) for narrow endemic and for nonendemic species.

Due to their influence on microclimatic and edaphic variation in many Mediterranean-type regions across the world (Rivas-Martínez et al. 1997; Hopper and Gioia 2004), elevation gradients are considered to be one of the most decisive factors shaping the spatial patterns of species diversity (Lomolino 2001; Wang et al. 2003; Kruckeberg 2004; Sánchez-González and López-Mata 2005). Although soil characteristics have been addressed in depth in the Mediterranean Cape and SW Australia, comparatively much more emphasis has been placed on geomorphological effects (elevation, geographical isolation, and climatic refugia) when attempting to explain plant biodiversity in the Mediterranean Basin (Heywood 1960; Médail and Quézel 1999; Petit et al. 2003; Körner 2007). Thus, geomorphological complexity determines an environmental mosaic that allows for the coexistence of species with disparate ecological requirements within a small area, which in part explains the high rates of Mediterranean biodiversity relative to other temperate climatic regions.

Edaphic profiles and spatial changes in soil properties have also been singled out as key drivers of beta diversity in Mediterranean-type biomes (Cowling et al. 1992; Hopper and Gioia 2004). This plant diversity–substrate relationship is particularly relevant in Mediterranean ecosystems given the high incidence of stressful soils such as lithosols (Pekin et al. 2012). Oyonarte et al. (2008) found that lithology was the main driver of the chemical properties of Mediterranean mountain soils (pH, texture, exchange complex, and free oxides). Chemically stressed soils are even harsher for plants in Mediterranean-type climate and promote ecological differentiation and endemism (Cowling and Holmes 1992; Kruckeberg 2004). Finally, spatial isolation and a limited amount of species-dispersal between ecoregions may also explain species turnover and generate the so-called patterns of distance-decay (Condit et al. 2002; Qian et al. 2005; Keil et al. 2012; Kristiansen et al. 2012), as predicted by the neutral theory of biodiversity (Hubbell 2001).

**Figure 1:**
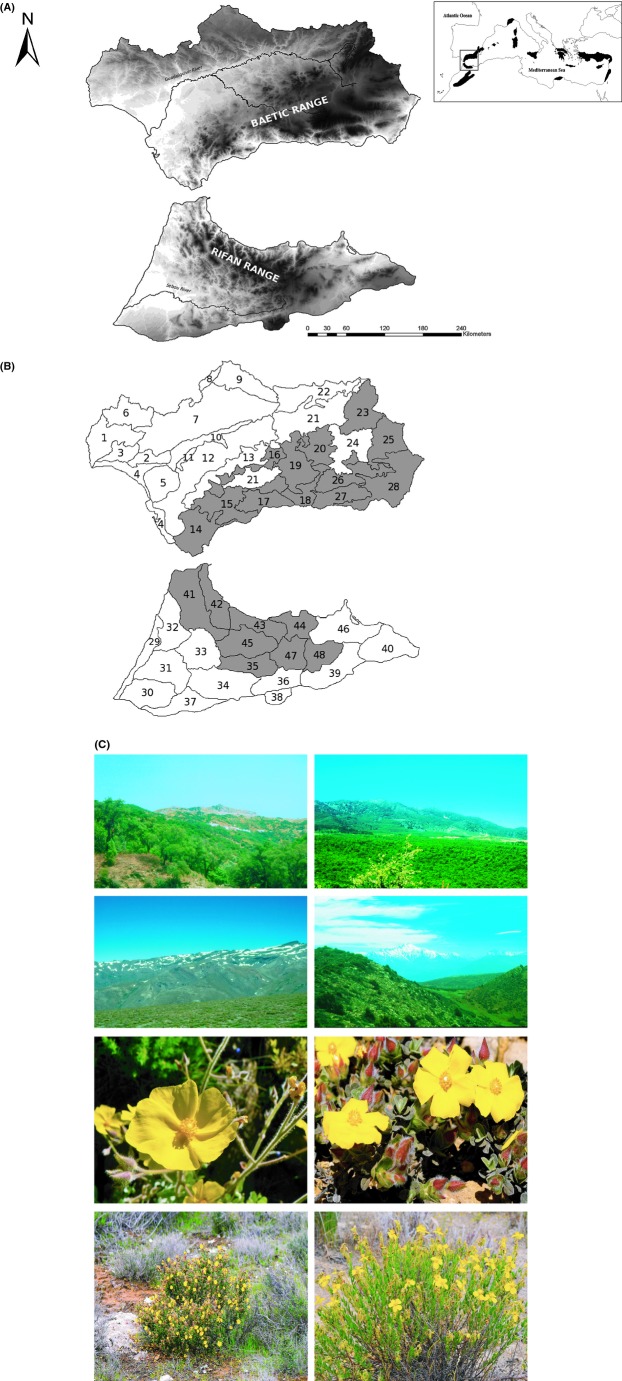
Location of the study region within the Mediterranean Basin. (A) Map of Andalusia (southern Iberian Peninsula) and northern Morocco (NW Africa), showing the position of the Baetic and Rifan ranges. The inset shows the Mediterranean Basin hotspots according to Médail and Quézel (1997) (modified from Médail and Quézel 1999). (B) Map showing ecoregions within Andalusia and northern Morocco (after Valdés et al. 1987, 2002; Blanca et al. 2009). The highlighted ecoregions correspond to those that match the mountain ranges forming the Baetic-Rifan hotspot. The numbers correspond to the names of ecoregions as in Appendix S1. (C) Top pictures represent typical landscapes of woodlands and chamaephyte highlands respectively in Andalusia (top-left and bottom-left corners) and North Morocco (top-right and bottom-right corners). Pictures by Juan Arroyo Marín. Bottom pictures represent two closely related pairs of taxa (Cistaceae) distributed on southern Iberian Peninsula and northern Morocco that are found under contrasting soil conditions (see Appendix S2 for references of molecular phylogenies of implied taxa). From left to right and top to bottom: *Halimium atriplicifolium* subsp. *atriplicifolium* (serpentines), *Halimium lasianthum* subsp. *lasianthum* (sandstones), *Helianthemum alypoides* (gypsum outcrops) and *Helianthemum polygonoides* (saline soils). Pictures by Abelardo Aparicio Martínez.

Within the Mediterranean Basin hotspot, most of the plant species richness (and thus a large part of the narrow Mediterranean endemism) is concentrated in the Iberian Peninsula and north-west Africa, as well as in the Balkan and Anatolian Peninsulas (Médail and Quézel 1997). Within the former two regions, plant biodiversity is to a large extent confined to the Baetic (S Iberian Peninsula) and Rifan (NW Africa) mountain ranges (Fig. 1) (Médail and Quézel 1999). Botanists have long been aware of the floristic similarities between the southern Iberian Peninsula and North Africa (Valdés 1991; Médail and Quézel 1997). Therefore, these regions are of particular interest given that they are considered to share characteristics despite being located on two different tectonic plates separated by the Strait of Gibraltar (Ajbilou et al. 2006). However, quantitative analyses have only been carried out in certain regions of the Baetic-Rifan hotspot (Mota et al. 2002; Valdés et al. 2006) or restricted to particular groups (Medina-Cazorla et al. 2010).

In this article, we aim to (1) determine the affinities between the floras of Andalusia and northern Morocco and (2) disentangle the environmental and geographical drivers of alpha and beta diversity within endemic and nonendemic plant assemblages in the Baetic-Rifan mountain ranges. Ultimately, our overall goal is to use the Baetic-Rifan hotspot as a model to improve our understanding of how environmental heterogeneity structures patterns of plant biodiversity in a Mediterranean range.

## Methods

### Study area

Andalusia and northern Morocco are geomorphologically heterogeneous areas that are characterized by high mountain ranges (e.g., Baetic-Rifan complex) surrounded by extensive lowlands of alluvial origin and shaped by the rivers Guadalquivir (Andalusia) and Sebou (northern Morocco) (see Fig. 1).

The Baetic and Rifan ranges share a common geological history and form part of the Alboran tectonic microplate located between the Eurasian and the African plates (Rosenbaum et al. 2002). The Baetic-Rifan range is rugged and rises to over 3400 m a.s.l. (i.e., Sierra Nevada) and boasts a heterogeneous lithology with substrata that include siliceous metamorphic outcrops, Oligo-Miocene sandstones, peridotites, limestones, dolomites, and marbles. This geomorphological complexity yields a wide range of climatic variation in terms of both temperature and precipitation, albeit always within the context of Mediterranean-type climate (see Appendix S1 in Supporting Information for details).

### Floristic datasets

We compiled an exhaustive distribution dataset for all the vascular plant species and subspecies occurring in Andalusia and northern Morocco based on the catalogs elaborated by Valdés et al. (1987) and Blanca et al. (2009) for Andalusia (updated whenever necessary with floristic studies published in peer-reviewed journals), and by Valdés et al. (2002) for northern Morocco. We followed nomenclatural criterion in Blanca et al. (2009). When different subspecies were recognized for a given species in Blanca et al. (2009) and not in Valdés et al. (2002) or Valdés et al. (1987), we used the information regarding the overall distribution of taxa in Blanca et al. (2009) and Castroviejo (1986) to assign the corresponding subspecies. When it was not possible due to overlapping subspecies ranges, we considered subspecies in Blanca et al. (2009) as a single species. We used the 48 ecoregions recognized in these sources to estimate geographical ranges of taxa based on their presence/absence in each ecoregion (Fig. 1B). An ecoregion is a territory characterized by the existence of homogeneous climatic, topographical, and geological features (Valdés et al. 2002). A territorial subdivision of this type, based on natural physiographic boundaries, has been used in a number of recent studies (e.g., Veech and Crist 2007) and is appropriate for different analytical methods. Note that the delimitation of an ecoregion does not imply that it has a unique flora.

We removed all non-native taxa from the dataset, as well as those taxa whose presence in the study area are doubtful or whose distribution range within the former is little known. We neither considered doubtful local citations. Despite their putative importance in some regions and relevance for descriptions of plant biodiversity, hybrids were not considered given the lack of comprehensive information about their constancy. For taxa lacking detailed distribution information, we searched for confirmed citations in the databases of the Spain Plant Information System (Anthos 2012) and the Global Biodiversity Information Facilities (GBIF 2012). The Algeciras–Aljibe and Grazalema–Ronda ecoregions were merged due to their small size and the high environmental resemblance between Algeciras and Grazalema (from Valdés et al. 1987) and Aljibe and Ronda (from Blanca et al. 2009), respectively. The ecoregions corresponding to the Baetic-Rifan range are unambiguously assigned in the source floras given that they match well the mountain ranges that form this hotspot. Despite ecoregions 21 and 24 are adjacent to those forming the Baetic range (see Fig. 1), they were not considered as Baetic ecoregions because they do not include high mountain areas, but sedimentary lowlands (i.e., ecoregion 21) or plateaus (i.e., ecoregion 24). Although Baetic mountains expand somewhat beyond the bounds of Andalusia through some adjacent mountainous areas in SE Iberian Peninsula, we did not include them in the analyses because the floristic knowledge regarding the easternmost range is not complete. However, this study covers more than 90% of the surface of the Baetic-Rifan hotspot.

### Endemic ranges

We split our floristic dataset into two elements according to their general distribution range: the first included taxa whose distributions are restricted to the Baetic-Rifan hotspot (hereafter, endemic element), while the second included all taxa with a broader distribution (hereafter, nonendemic element). To assess species’ ranges, we followed Castroviejo (1986), Valdés et al. (2002), Fennane and Ibn Tattou (2005), Blanca et al. (2009), and Ibn Tattou and Fennane (2009).

### Environmental variables

To describe climatic and altitudinal variation between ecoregions and to use them to depict the influence of the environment on biodiversity patterns, we used maximum-resolution rasters from the WorldClim database (Hijmans et al. 2005). We took a monthly value of maximum, minimum and mean temperatures, total precipitation, and elevation for each 1 km^2^ cell in the study area and then extracted the monthly means for each ecoregion. Subsequently, we used these scores to construct 22 new variables relating to temperature, precipitation, and elevation (see Table 1). We used the same climate layers from the WorldClim database to spatially model the current climate of Andalusian and North Moroccan areas, in order to avoid methodological biases due to sampling errors. Other putatively interesting energy-related variables were not equally available for Andalusia and North Morocco. However, many of the climatic variables had a high degree of collinearity, as expected for most of energy-related variables, with those used in the analyses. We also made use of a lithological map (scale 1:100,000) of Andalusia (Jordán 2000) in which 162 different categories of substrata were identified for the Baetic ecoregions.

**Table 1:** Set of climatic and altitudinal variables considered in the BIO-ENV analysis.

Code	Variable
1	Mean of monthly mean temperature
2	Variation of monthly mean temperature (SD)
3	Range of monthly temperature
4	Total monthly precipitation
5	Variation of monthly precipitation (SD)
6	Range of monthly precipitation
7	Mean of monthly maximum temperature
8	Variation of monthly maximum temperature (SD)
9	Mean of monthly minimum temperature
10	Variation of monthly minimum temperature (SD)
11	Precipitation of the driest month
12	Precipitation of the wettest month
13	Maximum temperature of the warmest month
14	Minimum temperature of the coldest month
15	Mean precipitation of driest annual quarter
16	Mean precipitation of wettest annual quarter
17	Mean temperature of coldest annual quarter
18	Mean temperature of warmest annual quarter
E1	Mean elevation
E2	Maximum elevation
E3	Minimum elevation
E4	Variation of elevation (SD)

The geometrical irregularity of the ecoregions in the study area makes single coordinate locations useless for the calculation of geographical distances. To solve this problem, the study area was divided into 1-km^2^ cells and the Euclidean distance was calculated between all pairwise cell comparisons for each pair of ecoregions. The Euclidean distances separating any two ecoregions were defined by the probability density of their constituent pairwise cell distances. We used the mean value of each probability density distribution as an estimate of the geographical distance between ecoregions (Euclidean).

### Statistical analysis

Floristic affinities between Andalusia and northern Morocco were assessed by conducting a hierarchical cluster analysis (HCA) based on the distribution of the vascular flora in the ecoregions of Andalusia and northern Morocco. The Sørensen similarity index was used as a distance function (1–Sørensen similarity index), while the unweighted pair-group method with an arithmetic mean (UPGMA) was used as an agglomerative algorithm to construct a rooted dendrogram. We measured how well the resulting clusters were supported by the data using a multiscale bootstrap resampling (*N* = 10,000) (Shimodaira 2004). We also conducted a detrended correspondence analysis (DCA) to examine further the biogeographical relationships between ecoregions in a multidimensional space.

To identify the drivers of alpha diversity in the Baetic-Rifan ecoregions, we explored their correlations with environmental (temperature and precipitation) and altitudinal variables. Given that both sets of variables had a high degree of collinearity, we conducted a principal components analysis (PCA) of all the environmental and altitudinal variables to reduce their dimensionality. Then, we fitted single linear models to both the endemic and nonendemic alpha diversity against each of the first principal components of environmental and altitudinal variations. We also regressed alpha diversity to the area occupied by each ecoregion.

Endemic and nonendemic compositional beta diversity matrices were calculated using the Sørensen similarity index as a dissimilarity measure (1–Sørensen similarity index) between the plant assemblages of the ecoregions. We constructed two additional dissimilarity matrices that only included the Baetic ecoregions to analyze the correlation between lithological features and plant assemblages. Unfortunately, we had to restrict this analysis to the Baetic range, because lithological information from North Morocco is scarcer, more incomplete, or not directly comparable with that available for Andalusia, which prevented us to further extend our conclusions to the whole Baetic-Rifan hotspot.

We used the BIO-ENV function (Clarke and Ainsworth 1993) to identify the subsets of climatic variables that produced the highest (nonparametric) correlation with each compositional beta diversity matrix. These optimal subsets were used to calculate the climatic distance matrices (Euclidean) between all pairs of ecoregions. We carried out the same procedure for the altitudinal variables. Finally, a lithological distance matrix using the Bray-Curtis dissimilarity index was constructed from the relative surface area occupied by each of the different categories on the lithological map.

For each compositional beta diversity matrix, we applied a multiple semi-partial regression on distance matrices analysis (Lichstein 2007) by relating plant assemblage dissimilarities to climatic, altitudinal, lithological, and geographical distance matrices. We partitioned the variance in compositional beta diversity matrices between single contributions from each explanatory variable, the covariation between them and the unexplained variance (Legendre and Legendre 1998). The significance for the regression coefficients was tested by generating 1000 permutations in the dataset. By resampling (*n* = 1000 iterations) with 667 replacement taxa (the size of the Baetic-Rifan endemic dataset) from the Baetic-Rifan nonendemic dataset (3384 taxa), we were also able to test whether or not the observed differences in species numbers between endemic and nonendemic elements might affect the output of the analysis. Finally, we calculated a distance matrix for each resampled matrix and regressed each to the original matrix (test significance conducted by permutations; *n* = 1000).

To ensure that the results were not biased by an a priori selection of Baetic-Rifan ecoregions, we conducted a subanalysis of beta diversity using only high-elevation ecoregions. First, we visually explored the cell-value distribution of the digital elevation model within each ecoregion. Then, we removed the nine ecoregions with either no significant fraction of their area over 1500 m or with most of their surface area below 500 m.

We used the R packages *vegan* (Dixon 2003) and *ecodist* (Goslee and Urban 2007) for the BIO-ENV and multiple regression on distance matrix analyses, respectively, and the *pvclust* package (Suzuki and Shimodaira 2006) for the hierarchical cluster analysis.

## Results

### Floristic datasets

Our input dataset consisted of 4450 native vascular plant species and subspecies from Andalusia and northern Morocco. The vast majority of species (91%) are present in the ecoregions assigned to the Baetic-Rifan hotspot. Of this Baetic-Rifan pool, 667 taxa are endemic (16.5%) and 3384 nonendemic. Of the endemic element, 485 taxa are confined exclusively to the Baetic range, while 129 are endemic to the Rifan mountains; in all, 53 endemic species are found in both ranges.

### Floristic affinities between Andalusia and northern Morocco

**Figure 2:**
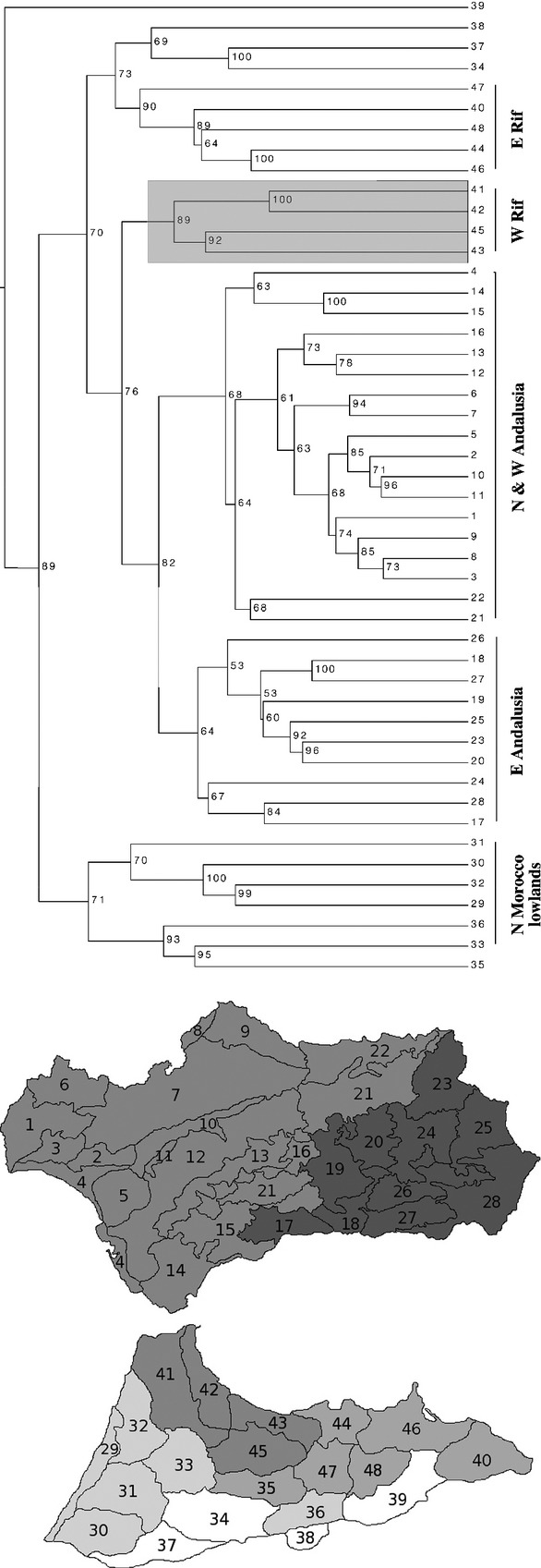
Hierarchical cluster analysis (UPGMA) representation showing the floristic relationship between the ecoregions of Andalusia and northern Morocco. Node numbers are Approximate Unbiased *P*-values in percentage. The highlighted clade represents the West Rifan ecoregions, which are grouped together in Andalusian clusters. For ecoregions names see Appendix S1.

The hierarchical cluster analysis showed that the plant assemblages of the Rifan mountain ecoregions and a number of other ecoregions at the southern boundary of northern Morocco (i.e., ecoregions 34, 37, and 38) are consistently (89% bootstrap support) more similar to the Andalusian ecoregions than to any other northern Morocco ecoregion (Fig. 2). Furthermore, plant assemblages of the ecoregions located at the western end of the Rifan mountains form alone a quite robust cluster (89% bootstrap support) that is closely related to all the Andalusian assemblages (76% bootstrap support).

**Figure 3:**
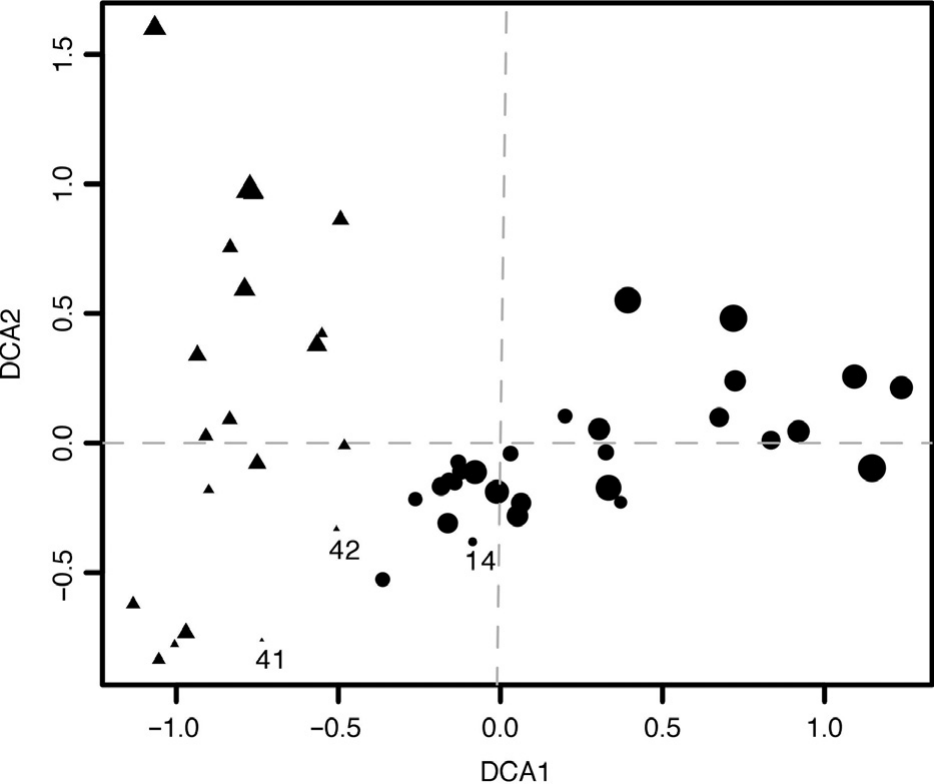
Detrended correspondence analysis (DCA) showing biogeographical relationships of ecoregions of Andalusia and northern Morocco. Circles represent Andalusian ecoregions, whereas triangles represent northern Morocco ecoregions. The size of the symbols is proportional to the ln of the geographic distance from the Strait of Gibraltar. Labelled points represent the ecoregions closer to the shores of the Strait of Gibraltar.

In contrast to the northern Moroccan ecoregions, which were spread along the second axis, the first axis of the DCA discriminated most of the Andalusian ecoregions (especially those in eastern Andalusia) from all others (Fig. 3). This second axis also defined the eastern Rifan ecoregions and the ecoregions around the Strait of Gibraltar as very different groups.

### Alpha and beta diversity in the Baetic-Rifan range

**Figure 4:**
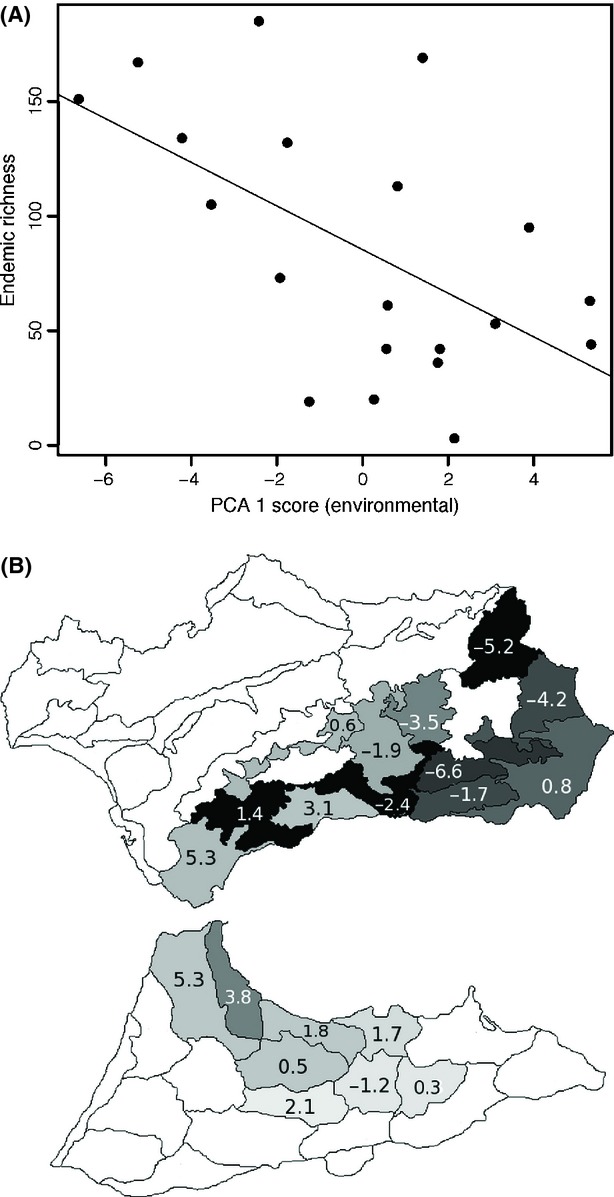
(A) Scatter plot of endemic alpha diversity recorded in Baetic-Rifan ecoregions against the scores of the first axis of the principal components analysis (PCA) for environmental variables. The solid line represents the fitted values from the linear model. (B) Map of endemic alpha diversity. The grey scale is proportional to endemic richness (maximum value in black). The numbers represent the scores of the PCA 1 component. Negative values are related to high values of precipitation in summer and to high temperature oscillation. Positive values are related to high minimum and high mean temperature values.

The first two eigenvalues of the PCA accounted for 62 and 67% of the accumulated variance in the environmental and altitudinal variables, respectively (see Table 3 for PCA scores). Nonendemic alpha diversity had a nonsignificant relationship with both the environmental and altitudinal variables (Adjusted R^2^ = −0.05, *P* = 0.85; Adjusted R^2^ = −0.05, *P* = 0.84), whereas the endemic alpha diversity was explained relatively well by the environmental variables (Adjusted R^2^ = 0.28, *P* < 0.01) but only poorly by the altitudinal variables (Adjusted R^2^ = 0.14, *P* = 0.05). Overall, greater endemic richness was mainly related to higher precipitation levels during the dry season and greater temperature oscillation, whereas lower endemic richness was related to both higher minimum and higher mean temperature values (Fig. 4). In no case, did the area occupied by each ecoregion explain the alpha diversity (Adjusted R^2^ = 0.07 and −0.02, *P* = 0.14 and 0.46 for nonendemic and endemic richness, respectively).

Different sets of climatic and altitudinal variables were selected to explain the diversity patterns for each floristic element (endemic and nonendemic). Minimum elevation was consistently included for both endemic and nonendemic floristic elements in the Baetic-Rifan analysis, whereas mean elevation was only selected in the analysis restricted to the Baetic range. The total monthly precipitation, the mean monthly maximum temperature, and the variation in the monthly minimum temperature (SD) were also included for both elements in the Baetic-Rifan analysis; on the other hand, the mean of the monthly mean temperature and the total monthly precipitation were included in the analysis restricted to the Baetic range (Table 2).

**Table 2:** Set of climatic and altitudinal variables that produced the highest nonparametric correlations for each floristic element (E, endemic; NE, nonendemic) according to BIO-ENV analysis.

Region	Climatic variables		Altitudinal variables		Climatic correlation		Altitudinal correlation	
			
	E	NE	E	NE	E	NE	E	NE
Baetic-Rifan	4, 7, 10	4, 7, 10, 13	E3	E3	0.45	0.44	0.07	0.13
Baetic	1, 4, 9, 13, 17	1, 2, 3, 4, 5, 7, 15, 18	E1	E1	0.88	0.91	0.69	0.76

**Figure 5:**
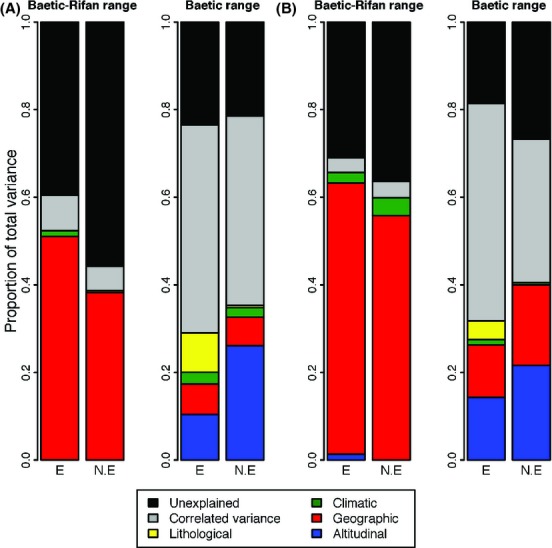
Proportion of the variance of the compositional beta diversity explained in each floristic element by climatic, lithological, altitudinal and geographic distances, correlated variation, along with the remaining unexplained part. (A) All Baetic-Rifan ecoregions. (B) High-elevation (> 1500 m) Baetic-Rifan ecoregions. (E, endemic; NE, nonendemic).

When all the Baetic and Rifan ecoregions were pooled, 60% (*P* < 0.001) and 44% (*P* < 0.001) of the total variance of the beta diversity was explained for endemic and nonendemic plant elements, respectively. Of all variables, geographical distance contributed by far the most to the explained variance, whereas both altitudinal and climatic variables together explained less than 2% in each case (Fig. 5A).

In all, 77% (*P* < 0.001) and 79% (*P* < 0.001) of the beta diversity in the Baetic ecoregions was explained for endemic and nonendemic elements, respectively. Altitudinal differences were by far the most significant variable for nonendemic elements and accounted for 74% of the variance explained by single factors. By contrast, the single contributions of lithological and altitudinal variables with regard to endemic plant assemblages were very similar and accounted for 31% and 36% of the variance explained by single contributions, respectively. Single climatic and geographical contributions were lower and very similar for both floristic datasets. In addition, a high fraction of the total variance was explained by the correlated variation between explanatory matrices in the analysis restricted to the Baetic dataset (Fig. 5A).

The beta diversity subanalysis between the high-elevation ecoregions of either the whole hotspot or of just the Baetic range mirrors the pattern observed in the complete analysis, differing only in a quantitative sense (Fig. 5B). Differences in the variance explained by the single contribution of the altitudinal distance between endemic and nonendemic elements lessened as a consequence of a loss in altitudinal variability. Similarly, there was a significant reduction in variance explained by lithology, probably due to the exclusion of certain low-elevation ecoregions (i.e., Aljibe, 14) that include particular outcrops with many endemic species (Ojeda et al. 2000). Overall, the correlations of the resampled matrices with the original matrix were high (mean Adjusted R^2^ = 0.96, SD = 0.009) and significant (mean *P*-value < 0.001, SD = 0.00), thereby confirming that results are not driven by the differences in species number between the two floristic elements.

## Discussion

Albeit still lacking in number, the availability of comprehensive taxonomic and distribution information for biodiversity hotspots offers an excellent opportunity for quantifying the extent and uniqueness of biodiversity and for unraveling the drivers of biodiversity distribution. In this respect – and as we assess quantitatively in this study – the Baetic-Rifan hotspot reveals interesting patterns such as the key role of substrate and geomorphology in shaping species diversity and spatial turnover in Mediterranean hotspots (Médail and Quézel 1997; Mota et al. 2002).

### Geographical structure of floristic diversity

The hierarchical cluster analysis of plant assemblages in the ecoregions of Andalusia and northern Morocco reveals the existence of remarkable floristic links between these two regions (Valdés 1991; Galán de Mera and Vicente Orellana 1997; Marañón et al. 1999). In contrast to the Baetic Cordillera, which seems to possess a certain degree of floristic unity, the plant assemblages of the West Rifan mountains seem to be more related to the Andalusian flora than to that of the East Rifan mountains and other nearby ecoregions. Furthermore, plant assemblages of the Rifan mountain range as a whole were consistently separated from that of the northern Morocco lowlands in the clustering analyses, thereby demonstrating their affinities to the Andalusian flora. Therefore, the biogeographical heterogeneity of northern Morocco contrasts with that of its Iberian counterpart, despite the poorer species richness and smaller surface area of the former. The DCA indicated that an overall floristic similarity gradient exists from the ecoregions in the west around the Strait of Gibraltar to the eastern end of the territory (Fig. 3). Both current and past conditions may be responsible for this pattern. At present, the clearest ecogeographical pattern across the region is a strong climatic gradient, being in general wetter and milder in the west and drier and colder in the east (Rivas-Martínez et al. 1997). Additionally, the geographical proximity between Andalusia and Africa is also much more marked in the west than in the east, which may explain in part the pattern in floristic similarities observed there (see Lavergne et al. 2013 for a specific account at both sides of the Strait of Gibraltar).

The south-west European and north-west African tectonic plates have been intermittently connected over geological times. At the end of Miocene (5.96 Myr BP), the Strait of Gibraltar closed and the Mediterranean dried up (Duggen et al. 2003). This allowed for African-Iberian biological exchanges to take place during the Messinian, which have been registered in the Spanish mammalian record (Agustί et al. 2006) and inferred for some plants (Guzmán and Vargas 2005). Fluctuations in sea level during the Quaternary glacial and interglacial periods (Yokoyama et al. 2001) also seem to have played an important role in shaping the structure of genetic diversity in the populations (Rodríguez-Sánchez et al. 2008) and have thus driven speciation when migration was interrupted. Lavergne et al. (2013) found that the Strait of Gibraltar caused a bias in species migration between the two sides, and this interruption in the gene flow had the effect of triggering local speciation in certain families with low dispersal capabilities. These results support the hypothesis that the Strait of Gibraltar has acted in the past as a migration route for plants from both sides of this strait. Our findings reveal the imprint of this phenomenon and expose the fundamental role played by the Strait of Gibraltar in the structuring of plant assemblages in this Mediterranean biodiversity hotspot, evident above all in the high floristic similarity at its western end.

### Environmental correlates of alpha and beta diversity

The area occupied by each ecoregion was not a good predictor of alpha diversity, thereby indicating that surface area does not directly affect the observed diversity patterns (as could be expected given the species–area relationships). Despite the fact that previous studies of alpha diversity correlates in the Iberian Peninsula have shown that elevation was the most significant variable explaining species richness distribution (Lobo et al. 2001), we found that other environmental variables explained the endemic alpha diversity far better. Our findings suggest that climatic variables shape the spatial distribution of endemic plant richness in the Baetic-Rifan range. In Mediterranean ecosystems, productivity drastically decreases during the summer drought and, given that the ecoregions of high endemic richness are characterized by relatively high summer precipitation (see Table 3), it is probable that summer drought acts as an ecological limiting factor for speciation in ecoregions of low endemic richness (Anacker and Harrison 2012).

**Table 3:** Principal component analysis scores for the environmental variables (axis 1).

Variable	PC1 score
Total monthly precipitation	0.11
Variation of monthly precipitation (SD)	0.21
Range of monthly precipitation	0.21
Precipitation of the wettest month	0.18
Precipitation of the driest month	**−0.28**
Mean precipitation of wettest annual quarter	0.18
Mean precipitation of driest annual quarter	**−0.27**
Mean of monthly mean temperature	**0.27**
Variation of monthly mean temperature (SD)	**−0.26**
Range of monthly temperature	**−**0.24
Maximum temperature of the warmest month	**−**0.01
Minimum temperature of the coldest month	**0.28**
Mean of monthly maximum temperature	0.25
Variation of monthly maximum temperature (SD)	**−0.26**
Mean of monthly minimum temperature	**0.28**
Variation of monthly minimum temperature (SD)	**−0.26**
Mean temperature of warmest annual quarter	0.20
Mean temperature of coldest annual quarter	**0.29**

In bold, scores higher than 0.25 in absolute value.

Although its importance dropped drastically when we focused only on the Baetic ranges, geographical distance was by far the most important factor explaining beta diversity between ecoregions within the hotspot as a whole. The strong independent effect of geographical distance on plant assemblages at hotspot spatial scale indicates that limitations on dispersal (dispersal assembly) seem to govern the spatial distribution of plant diversity; nevertheless, at smaller scales, environmental sorting (niche assembly) is more important. Thus, it is probable that a substantial fraction of the flora of the Baetic range is missing from Rifan range areas with suitable environments (and vice versa) because the Atlantic Ocean and the Mediterranean Sea, which separate these mountainous ranges, act as an effective barrier preventing migration between these regions, at least in the easternmost range.

In the analysis restricted to the Baetic range, altitudinal differences between ecoregions seem to be the main factor explaining beta diversity patterns, albeit to differing degrees for endemic and for nonendemic taxa. The same applies to lithological differences, although they were a much better predictor for endemic species than for nonendemic taxa. These findings imply that diversity patterns of endemism do not necessarily mirror the diversity patterns of the regional pool in which endemic taxa are included (Whittaker et al. 2001; Vetaas and Grytnes 2002).

The great incidence of altitudinal distance on beta diversity patterns reported in our study agrees with traditional claims that orographic isolation plays an important role in plant diversification and endemism in the Mediterranean (Heywood 1960). Furthermore, both maximum elevation and the variation in elevation (SD) are well correlated within the Baetic-Rifan ecoregions (*r* = 0.72), indicating that environmental heterogeneity shaped by altitudinal gradients could play a key role in explaining beta diversity patterns in the Baetic ranges.

Without taking into account current environmental conditions, the importance of altitudinal differences as determinants of beta diversity can be in part linked to past historical scenarios (Cowling et al. 1996; Cowling and Lombard 2002). Historical factors such as past climate, postglacial recolonization, and habitat stability have been reported to be important determinants of both alpha (Svenning and Skov 2007; Svenning et al. 2009) and beta diversity (Graham et al. 2006; Dexter et al. 2012) patterns. Good phylogeographical and fossil evidence exist to suggest that the mountains of southern Spain were an important glacial refugium during the Pleistocene ice ages (Salvador et al. 2000; Carrión et al. 2003; Hampe and Petit 2005; Cheddadi et al. 2006; Médail and Diadema 2009) that allowed populations of more northerly distributed plant species to persist (see Blanca et al. 1998).

### The potential imprint of climate stability and limitations on dispersal

Local extinction rates at higher elevations increase during glacial–interglacial periods in response to shifts in climatic conditions. Nevertheless, the local rates of displacement in climatic conditions in the Baetic range during the Pleistocene glaciations were relatively low given its latitude and the influence of both the Atlantic Ocean and the Mediterranean Sea (Finlayson and Carrión 2007), thereby explaining population persistence in the Baetic range for both endemic and nonendemic taxa. Cowling and Lombard (2002) suggest that the greater climatic stability in the Western Cape Floristic Region during the Pleistocene explains this region's greater beta diversity compared with the Eastern Cape, where Pleistocene climates did not favor Cape lineages.

The overall low dispersal capacity of small-ranged taxa in Mediterranean biomes is well known (Cowling et al. 1994; McDonald et al. 1995; Melendo et al. 2003; Hopper and Gioia 2004). In a comparative study of two groups of western Mediterranean narrow endemic species and their corresponding congeneric nonendemic taxa, Lavergne et al. (2004) found that narrow endemics occur in habitats with greater rock cover, produce fewer seeds and smaller flowers, and have lower pollen/ovule ratios. These results suggest that local persistence rather than wide dispersal could be the dominant perpetuation strategy in Mediterranean narrow-ranged endemic plants. Thus, it is reasonable to assume that Baetic endemic plants have evolved in a very close relationship with the substrates of the areas in which they normally occur. Substrate conditions have been shown to act as selective forces driving diversification (Rajakaruna 2004). The large number of cases in the study area in which closely related taxa are found living under contrasting soil conditions can be interpreted as the imprint of this selection process caused by an adaptation to different edaphic conditions (Gutiérrez et al. 2004 and see Appendix S2 in Supporting Information for further examples). The notable incidence of lithological differences between ecoregions provides an even better explanation of endemic beta diversity and suggests that local adaptation to lithological conditions and speciation is a key mechanism in the shaping of biodiversity in this Mediterranean hotspot. Baetic range performs as an archipelago of mountains, isolated between them by lower areas of different substrata, which could act as biogeographical barriers preventing gene flow between highlands populations and driving differentiation (Hernández Bermejo and Sainz Ollero 1984). However, any full account of these processes still should consider the phylogenetic relationships between the taxa involved (Anacker et al. 2011).

## Concluding Remarks

In conclusion, plant assemblages in the Rifan mountains overall resemble the flora of Andalusia more than that of other northern Morocco neighboring ecoregions (and above all to those of the western Rifan mountains). The Baetic-Rifan ecoregions of high endemic richness are characterized by relatively high precipitation levels during the summer, which suggest that drought could be acting as an ecological factor limiting speciation in ecoregions of low endemic richness. When considering the whole Baetic-Rifan region, geographical distance is the main factor explaining variation in beta diversity in plant assemblages (ecoregions), even though the importance of this factor falls drastically at the scale of the Baetic range. Differences in elevation are a further important factor explaining floristic similarities in the Baetic range, especially in regard to nonendemic assemblages. By contrast, lithological features explain as much variance as elevation does for endemic assemblages and are of very little importance in nonendemic assemblages.

Diversity analyses using species and subspecies that do not take into account phylogenetic relationships suffer from severe limitations when searching for the underlying factors behind the origins and maintenance of biodiversity. For instance, excessive importance may be given to a local adaptation to a specific substrate if all the species in a clade are closely associated with that substrate. Future comparative studies of the Baetic-Rifan biodiversity hotspot should incorporate improved assessments of spatial diversity distribution using phylogenetic analysis (beta phylodiversity), which will help determine how contemporary and historical factors interact in the structuring of plant assemblages in the Baetic-Rifan and other biodiversity hotspots.

## Supporting Information

**Appendix S1.** Climatic variables, chorological assignation, and species richness.

**Appendix S2.** Some examples of close relative edaphisms within the Baetic range.
