# Disentangling environmental correlates of vascular plant biodiversity in a Mediterranean hotspot

**DOI:** 10.1002/ece3.904

**Published:** 2013-11-25

**Authors:** Rafael Molina-Venegas, Abelardo Aparicio, Francisco José Pina, Benito Valdés, Juan Arroyo

*Ecology and Evolution* 2013; 3(11): 3879–3894

doi: 10.1002/ece3.762

An error concerning data analyses was made by the authors in the original version of this article. In this article, climatic variables were not standardized prior to the computation of Euclidean distances between ecoregions. After correction for this issue, the interpretations are modified. The exclusive role of altitude variation should be neglected. Most of the altitudinal variation is now correlated variation between the factors considered, as detailed in the new Figure 5 (gray bars). The exception to this pattern is seen in the endemic component in the highest elevation range (Fig. 5B) where altitude explains a portion of the variability. A new version of Figure 5 is provided below.

**Figure 5:**
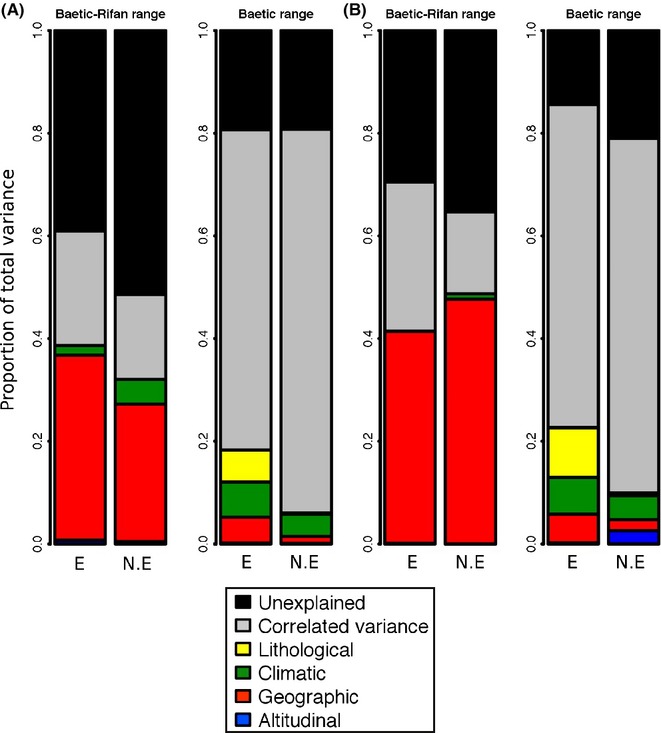
Proportion of the variance of the compositional beta diversity explained in each floristic element by climatic, lithological, altitudinal, and geographic distances, correlated variation, along with the remaining unexplained part. (A) All Baetic-Rifan ecoregions. (B) High-elevation (>1500 m) Baetic-Rifan ecoregions. (E, endemic; NE, nonendemic).

Due to the above changes, portions of the article regarding the methods and results tied to Figure 5 have been updated. The changes occur on pages 1, 6, 7, 9, 11, and 12.

