# Supplementary material for: Disentangling environmental correlates of vascular plant biodiversity in a Mediterranean hotspot
Source: Ecol Evol. 2013 Sep 15;3(11):3879–94. doi: 10.1002/ece3.762 (PMC3810881; doi:10.1002/ece3.762)
Supplement: Supplementary file 2 [file ece30003-3879-SD2.doc]

**APPENDIX S2.** Some examples of closely related pairs of taxa that are found under contrasting soil conditions within the Baetic range, following nomenclatural criteria in Blanca *et al*. (2009).

| Family | Pair of taxa | Substrate affinity |
| --- | --- | --- |
| Asteraceae | *Leucanthemopsis pallida* subsp. *pallida*  *Leucanthemopsis pallida* subsp. *spathulifolia* | Siliceous  Calcicole |
| Asteraceae * | *Centaurea carratracensis*  *Centaurea gadorensis* | Serpentines  Calcareous |
| Brassicaceae * | *Brassica repanda* subsp. *confusa*  *Brassica repanda* subsp. *latisiliqua* | Limestones  Dolomites |
| Brassicaceae | *Draba hispanica* subsp. *hispanica*  *Draba hispanica* subsp. *laderoi* | Limestones  Schists |
| Brassicaceae | *Erysimum baeticum* subsp. *baeticum*  *Erysimum baeticum* subsp. *bastetanum* | Siliceous  Basophilic |
| Brassicaceae | *Iberis carnosa* subsp. *embergeri*  *Iberis carnosa* subsp. *granatensis* | Schists  Limestones and dolomites |
| Caryophyllaceae | *Arenaria tetraquetra* subsp. *amabilis*  *Arenaria tetraquetra* subsp. *murcica* | Siliceous  Limestones |
| Caryophyllaceae | *Silene inaperta* subsp. *inaperta*  *Silene inaperta* subsp. *serpentinicola* | Limestones and Siliceous  Serpentines |
| Caryophyllaceae | *Silene psammitis* subsp. *psammitis*  *Silene psammitis* subsp. *lasiostyla* | Siliceous  Dolomites and serpentines |
| Cistaceae * | *Helianthemum alypoides*  *Helianthemum polygonoides* | Gypsum  Saline soils |
| Cistaceae * | *Halimium lasianthum* subsp. *lasianthum*  *Halimium atriplicifolium* subsp. *atriplicifolium* | Sandstones  Serpentines |
| Fabaceae * | *Anthyllis vulneraria* subsp. *reuteri*  *Anthyllis vulneraria* subsp. *pseudoarundana* | Limestones  Schists |
| Fabaceae * | *Cytisus scoparius* subsp. *scoparius*  *Cytisus scoparius* subsp. *reverchonii* | Siliceous  Calcareous |
| Plantaginaceae | *Linaria oblongifolia* subsp. *aragonensis*  *Linaria oblongifolia* subsp. *benitoi* | Basophilic  Siliceous |
| Plantaginaceae | *Linaria oligantha* subsp. *oligantha*  *Linaria oligantha* subsp. *valentina* | Gypsum  Limestones |
| Plumbaginaceae * | *Armeria filicaulis* subsp. *nevadensis*  *Armeria filicaulis* subsp. *trevenqueana* | Siliceous  Dolomites |
| Plumbaginaceae * | *Armeria villosa* subsp. *carratracensis*  *Armeria villosa* subsp. *villosa* | Serpentines  Limestones and dolomites |
| Poaceae | *Koeleria dasyphylla* subsp. *dasyphylla*  *Koeleria dasyphylla* subsp. *nevadensis* | Limestones  Siliceous |

* See references for molecular phylogenies of implied taxa.

**References**

Cubas, P., Pardo, C. & Tahiri H. (2002) Molecular approach to the phylogeny and systematics of *Cytisus* (Leguminosae) and related genera based on nucleotide sequences of nrDNA (ITS region) and cpDNA (trnL-trnF intergenic spacer). *Plant systematics and Evolution*, **233**, 223–242.

García-Jacas, N., Uysal, T., Romashchenko, K., Suárez-Santiago V.N., Ertugrul, K. & Susanna, A. (2006) *Centaurea* revisited: a molecular survey of the *Jacea* group. *Annals of Botany*, **98**, 741–753.

Gutiérrez, B., Aguilar, J. & Feliner, G. (2004) Morphometric and molecular evidence for taxonomic recognition of a new subspecies of *Armeria filicaulis* (Plumbaginaceae). *Anales del Jardín Botánico de Madrid*, **61**, 35–48.

Gutiérrez, B., Aguilar, J. & Feliner, G. (2006) Dispersal across southern Iberian refugia? Integrating RAPDs, sequence data and morphometrics in *Armeria* (Plumbaginaceae). *Folia Geobotanica*, **41**, 305–322.

Guzmán, B. & Vargas, P. (2005) Systematics, character evolution, and biogeography of *Cistus* L. (Cistaceae) based on ITS, trnL-trnF, and matK sequences. *Molecular Phylogenetics and Evolution*, **37**, 644–660.

Lega, M., Fior, S., Prosser, F., Bertolli, A., Li, M. & Varotto, C. (2012) Application of the unified species concept reveals distinct lineages for disjunct endemics of the *Brassica repanda* (Brassicaceae) complex. *Biological Journal of the Linnean Society*, **106**, 482–497.

Nanni, L., Ferradini, N., Taffetani, F. & Papa, R. (2004) Molecular phylogeny of *Anthyllis* spp. *Plant Biology*, **6**, 454–464.

Parejo-Farnés, C., Albaladejo, R.G., Arroyo, J. & Aparicio, A. (2013) A phylogenetic hypothesis for *Helianthemum* (Cistaceae) in the Iberian Peninsula. *Botanica complutensis* (in press).
